# Enzymatic activity of a recombinant β-1,4-endoglucanase from the Cotton Boll Weevil (*Anthonomus grandis*) aiming second generation ethanol production

**DOI:** 10.1038/s41598-019-56070-1

**Published:** 2019-12-20

**Authors:** Liz Nathalia Ibarra, Ana Nathalia Oliveira de Araújo Alves, José Dijair Antonino, Guilherme Souza Prado, Clidia Eduarda Moreira Pinto, Carlos Ricardo Soccol, Érico Augusto Rosas de Vasconcelos, Maria Fátima Grossi-de-Sa

**Affiliations:** 1 Universidade Federal do Paraná – UFPR, Curitiba, PR 81530-980 Brazil; 2 Embrapa Recursos Genéticos e Biotecnologia, Brasília, DF 70770-917 Brazil; 3 Universidade de Brasília – UnB, Biology Institute, Brasília, DF 70910-900 Brazil; 4 Universidade Federal Rural de Pernambuco – UFRPE, Recife-PE, 52171-900 Brazil; 5 Universidade Católica de Brasília – UCB, Brasília, DF 70790-160 Brazil; 6 Centro Universitário Euroamericano - UNIEURO, Brasilia, DF 70200-001 Brazil

**Keywords:** Biotechnology, Biochemistry

## Abstract

In the last years, the production of ethanol fuel has started to change with the introduction of second-generation ethanol (2 G Ethanol) in the energy sector. However, in Brazil, the process of obtaining 2 G ethanol did not reach a basic standard to achieve relevant and economically viable results. Several studies have currently been addressed to solve these issues. A critical stage in the bioethanol production is the deployment of efficient and stable enzymes to catalyze the saccharification step into the process of biomass conversion. The present study comprises a screening for genes coding for plant biomass degradation enzymes, followed by cloning a selected gene, addressing its heterologous expression, and characterizing enzymatic activity towards cellulose derived substrates, with a view to second-generation ethanol production. A cDNA database of the Cotton Boll Weevil, *Anthonomus grandis* (Coleoptera: Curculionidae), an insect that feeds on cotton plant biomass, was used as a source of plant biomass degradation enzyme genes. A larva and adult midgut-specific β-1,4-Endoglucanase-coding gene (*AgraGH45-1*) was cloned and expressed in the yeast *Pichia pastoris*. Its amino acid sequence, including the two catalytic domains, shares high identity with other Coleoptera Glycosyl Hydrolases from family 45 (GH45). AgraGH45-1 activity was detected in a Carboxymethylcellulose (CMC) and Hydroxyethylcellulose (HEC) degradation assay and the optimal conditions for enzymatic activity was pH 5.0 at 50 °C. When compared to commercial cellulase from *Aspergillus niger*, Agra GH45-1 was 1.3-fold more efficient to degrade HEC substrate. Together, these results show that AgraGH45-1 is a valid candidate to be engineered and be tested for 2 G ethanol production.

## Introduction

In the last 44 years, Brazilian fuel industry makes progress in the production of ethanol fuel. Sugar cane is the main raw material used in the country for ethanol production, which has been very successful in recent years^1,2^. Until not that long ago, the country’s production was limited to the fermentation of sugar cane molasses. However, since 2007, several 2 G ethanol research funding projects have been established, with the founding of new laboratories and research institutions. These projects came to fruition with the start of large-scale 2 G ethanol production, when in 2014, the first three commercial 2 G ethanol plants started operating^3^. Besides all scientific and technological foment, Brazilian 2 G ethanol industry faced some problems, such as enzymes cost, to keep sustainable activities and supply the market fuel^4^.

The conversion of plant cell wall polysaccharides into second-generation ethanol has been studied by different approaches, ranging from the use of genetically modified plants, which are developed for obtaining plant cell walls that are more sensitive to enzyme degradation^5,6^, up to engineering more efficient enzymes that are applied in chemical techniques for biofuel production^7,8^. Specific enzymes acting on the hydrolysis of β-glycosidic bonds in cellulosic biomass are still a limiting factor in this respect. While the production of cellulases has increased over the years, there has been a decrease in the costs of saccharification processes for the production of fermentable sugar^7,9^. Studies about enzymes production and stabilization methods are in progress, aiming to drop even more the cost for bioethanol production and other industrial applications, such as feed manufacture, laundry, and textile processing^10–12^. This bottleneck in the development of an efficient system for the production of second-generation ethanol underscores the needed for new enzymes that show high efficiency and stability, being consequently used in the conversion of biomass into fermentable products^13,14^.

Although microorganisms, such as bacteria and fungi, are the primary source of cellulolytic enzymes^15–17^, recently it has been found that invertebrates like insects and nematodes possess a whole arsenal of enzymes that degrade plant cell walls, such as glycosyl hydrolases (endoglucanases, polygalacturonases and xylanases) and other pectin-modifying enzymes^18–20^. Remarkably, it has been proposed that insects and nematodes have acquired these enzymes from bacteria and fungi through horizontal gene transfer with many independent events occurring along time^21^. It has been proposed that the acquisition of these enzymes has permitted insects, especially from the Coleoptera order, to gain an advantage efficiently exploring plant biomass^22^. Regarding insects, many of these enzymes have been validated *in vivo* at different systems such as insect cell lines^23^ and yeast, especially *Pichia pastoris* and *Saccharomyces cerevisae*^24,25^. Indeed, as far as we know, no microorganism had been engineered with any insect-derived enzyme other than an alpha-amylase from *Sitophilus oryzae* was used for simultaneous saccharification and fermentation (SSF) from raw starch aiming bioethanol production^26^. Moreover, insect genomes contain considerable amounts of genes coding for plant cell wall-degrading enzymes^27^, especially coleopteran insects from Curculionoidea and Chrysomeloidea superfamilies^23^. Several insects from the same family possess a number of these enzymes, such *Sitophilus oryzae*^28^, *Dendroctonus ponderosae* and *Hypotenemus hampei*^29^. Specifically, the Cotton Boll Weevil, *Anthonomus grandis*, (Coleoptera: Curculionidae), feeds on cotton plant biomass from floral bud and cotton boll during its life cycle, so its digestive plant cell wall-degrading enzymes are essential for its nutrition. We have published a transcriptome of *A. grandis*^30^, and it could represent an important source of genes coding to plant biomass degradation enzymes.

In this work, we report the cloning and heterologous expression of a *β-1,4-endoglucanase* gene specifically expressed in *A. grandis* larva and adult midgut (*AgraGH45-1)*. The gene was expressed in *P. pastoris* heterologous system and the recombinant enzyme was shown to be efficient to degrade Carboxymethylcellulose (CMC) and Hydroxyethylcellulose (HEC) at pH 5.0 and 50 °C. Here have we showed that AgraGH45-1 activity was 1,3-fold higher and more efficient to degrade HEC than commercial cellulase from *Aspergillus niger* at 50 °C. In addition, with temperatures ranging from 40 °C to 60 °C, the efficiency of AgraGH45-1 is not as affected as the one from commercial *A. niger* cellulase.

## Results and Discussion

In the Brazilian fuel industry, the process of obtaining 2 G ethanol did not reach a basic standard to conduct the production steps in order to achieve relevant and economically viable results. The lack of an agricultural and industrial system designed to make full use of sugarcane, biomass pretreatment, capital cost, pentose fermentation and enzyme cost are bottle necks that need to be overcome in order to keep sustainable industrial activity and supply Brazilian fuel market^4,31^. Currently, several researches are being developed to solve these problems, research focused on the 2 G ethanol production efficiency, economical processing of raw materials^32^, use of all fermentable fractions^33^, production of efficient and profitable enzymes^34,35^, among others. Here we shown, in a first step, the efforts to clone a *β-1,4-endoglucanase* gene from Cotton Boll Weevil . After that, a functional recombinant enzyme (AgraGH45-1) was produced and evaluated about its efficiency to catalyze cellulose derived substrate.

### Gene selection and AgraGH45-1 clone procedures for heterologous expression

A review of the main enzymes used in the plant biomass fermentation industry was conducted by mining through data on current protocols of plant biomass fermentation for the generation of bioenergy. The glucose production from plant biomass requires a serial action of at least three main enzymes from the Glycosyl Hydrolyse (GH) family: β-1,4-endoglucanases, cellobiohydrolases and β-glucosidases. A search for conserved nucleotide sequences coding for each one of these enzymes was performed under the Arthropoda taxon in NCBI-GenBank database. Conserved sequences were used as query in a search for similar sequences into *A. grandis* cDNA database. Subsequent analyses, considering the enzyme potential for cellulose degradation, applicability to plant biomass fermentation and BlastX hits e-value (< 1e^−30^), pointed to β-1,4-endoglucanase as the best target for cloning procedures. β-1,4-endoglucanases are crucial to break glycoside linkages that joins glucose residues in cellulose polymers, the first step to enzymatic cellulose degradation for obtaining the fermentable product, glucose^36^. However, to an efficient bioprocess, β-1,4-endoglucanases activity should be in synergism with other plant biomass degradation enzymes, encompassing an enzyme consortium for which substantial pH and heat stability are fundamental. The *A. grandis* β-1,4-endoglucanases gene cloned in this work was named *AgraGH45-1* and its enzyme product was assayed for pH and temperature efficiency.

**Figure 1:**
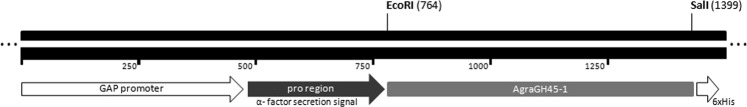
Schematic representation of the insertion of *A. grandis* β-1,4-endoglucanase gene (*AgraGH45-1*) into pGAPZα-B expression vector. *AgraGH45-1* was subcloned between *Eco*RI and *Sal*I restriction sites in frame with the α-factor secretion signal and under control of the *GAP* constitutive promoter.

The nucleotide sequence from *A_grandis_454_rep_c946* contig (Accession number: GABY01019746.1), comprising a *β-1,4-endoglucanase* putative gene (*AgraGH45-1*), was first used to design primers for cloning the whole sequence of *AgraGH45-1*, including its 3′ UTR by running a 3′ RACE-PCR assay (Table 1). Further, we subcloned the entire mature enzyme-coding sequence, without the signal peptide, (named AgraGH45-1 ΔSP, which comprises 654 bp that are translated to 217 amino acids residues). Then, the amplicon *AgraGH45-1* ΔSP was subcloned with restriction sites inserted on its 3′ and 5′ ends in order to allow its insertion into the pGAPZα-B vector in frame with the α-factor secretion signal and under control of the *GAP* constitutive promoter for heterologous expression in *P. pastoris* (Fig. 1). The subcloned expression vector was sequenced and confirmed as coding to a *β-1,4-endoglucanase* by BLAST based on GenBank-NCBI, confirming the sequence from *A. grandis* transcriptome.

**Table 1:** Primers used in RT-qPCR experiments.

Gene	Primer name	Primer Sequence (5′-3′)
*AgraGH45-1*	AgraGH45-1_qPCR_Fw	ATGTGACGAGCTCCCTACAGA
	AgraGH45-1_qPCR_Rv	TTCAGTTGGGCAGGTAATTTG
*GAPDH*	AgraGAPDH_qPCR_F	AGATCGTCGAGGGTCTGATG
	AgraGAPDH_qPCR_R	AAGGCGGGAATGACTTTACC
*β-Tubulin*	AgraBtub_qPCR_F	GGTTGCGACTGTTTACAAGG
	AgraBtub_qPCR_R	GCACCACCGAGTAAGTGTTC

### Insect transcription profile of AgraGH45-1

**Figure 2:**
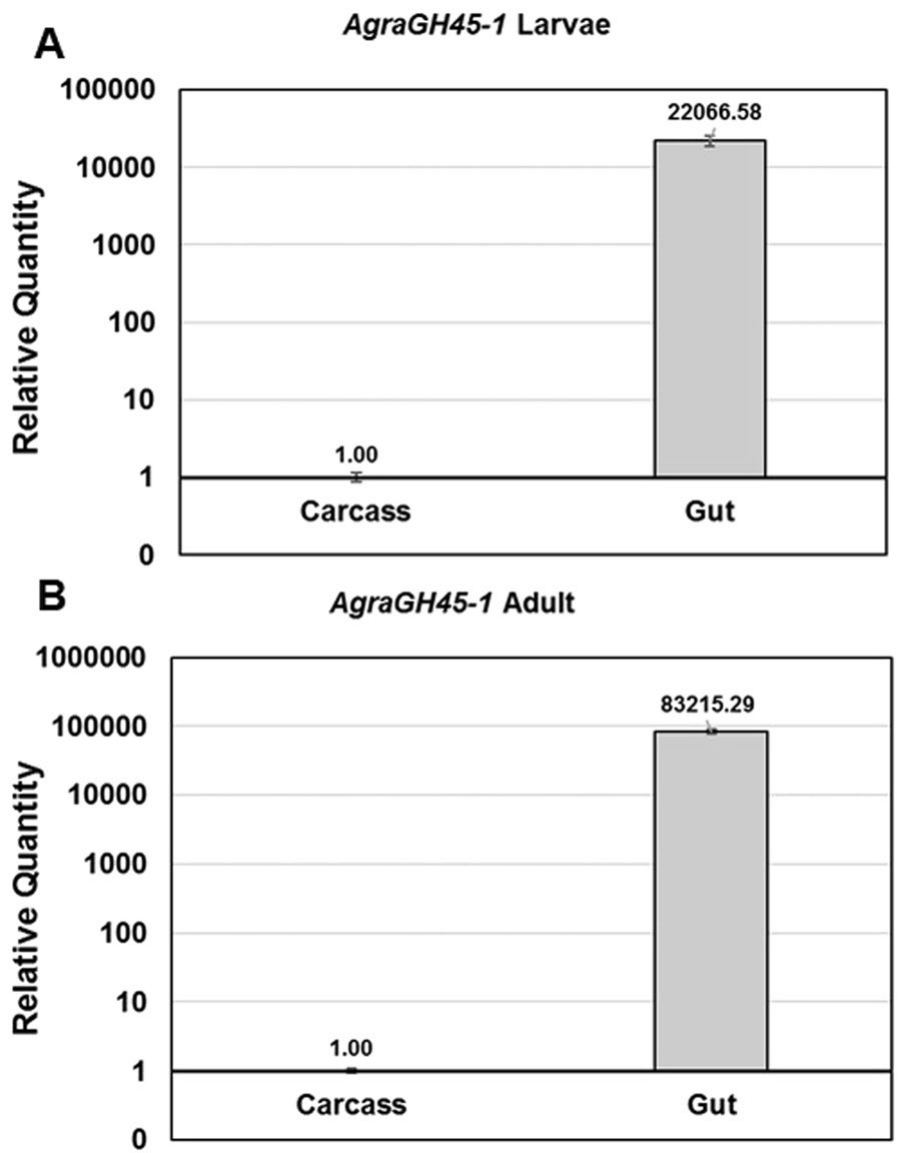
Relative quantification of *AgraGH45-1* in different *A. grandis* tissues. Quantification in larva (**A**) and adult (**B**) carcass and whole gut.

Initially, we have evaluated the transcript profile of *AgraGH45-1* in *A. grandis* adult and larval tissues, including midgut and carcass. The carcass comprises all other insect tissues without the intestine. In both stages, *AgraGH45-1* is far more expressed in the midgut than in carcass (Fig. 2A,B). These results, together with the presence of a predicted signal peptide, suggest that it can be a functional enzyme^19^. Indeed, coleopterans from families Curculionidae and Chrysomelidae have variable number of Glycosyl hydrolases in their genomes, including endoglucanases^19,23,37^ from families GH5, GH9, GH45 and GH48. However, functional validation *in vivo* is still necessary due to the special feature of non-functional enzymes may act as a decoy to compete with plant-derived enzyme inhibitors, as suggested by Kirsch and colleagues^20^, or even new function acquisition being specific to a different substrate^38^.

### AgraGH45-1 amino acids alignment with other endoglucanases

**Figure 3:**
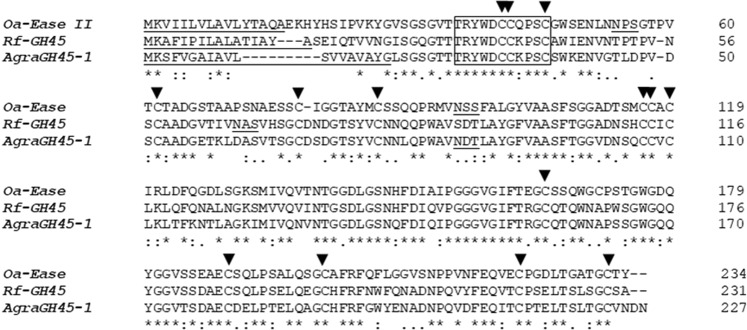
Alignment of the amino acid sequence of beetle β-1,4-endoglucanases. Signal peptide is underscored at N-terminus end. The conserved catalytic site, including catalytic aspartic acid (D), is shown in a solid box. Potential N-glycosylation sites are underlined below the alignment. Arrows indicate conserved cysteine residues.

Endoglucanases are organized into 13 Glycosyl Hydrolases (GH) families based on primary and tertiary structures similarities, substrate specificities, as well as catalytic mechanisms^39^. Such properties need to be appreciated when a recombinant enzyme is considered for industrial purposes. Plant biomass conversion process requires temperature and pH patterns in which both primary and tertiary enzyme structures need to keep its stability to improve enzyme activity. The predicted amino acid sequence of AgraGH45-1 shares a high identity level on the active site with other functional family 45 glycosyl hydrolases from beetles (Fig. 3), such as Rf-GH45, a *Rhynchophorus ferrugineus* β-1,4-Endoglucanase (74% whole amino acid identity) and Oa-EGase II, a β-1,4-Endoglucanase from *Oncideres albomarginata*, which structure was modeled by homology and suggests a polyphyletic origin for animal cellulases^27^. The conservation of tertiary structure in a recombinant β-1,4-Endoglucanase is also fundamental for enzymatic efficiency. Gao and colleagues have shown that a loop movement is needed to the catalytic reaction in a *Thielavia terrestris* β-1,4-Endoglucanase^40^, which shares the same catalytic mechanism than AgraGH45-1, where a aspartic acid (D), close to N-terminus (Fig. 3), serve as catalytic acid. The catalytic aspartic acid is conserved among AgraGH45-1, Rf-GH45 and Oa-EGase beetle β-1,4-Endoglucanases and shown in Fig. 3. Oa-EGase II shares 56% whole amino acid identity with AgraGH45-1. Besides all amino acids are conserved in the catalytic domains of the three beetle β-1,4-Endoglucanase, the sequences show slight differences between amino acids from glycosylation sites (Fig. 3). BLASTx results also reported a weak match against an *Aspergillus niger* Glycosyl Hydrolase (GH61), showing only 22% identity with AgraGH45-1.

### AgraGH45-1 heterologous expression in *P. pastoris*

**Figure 4:**
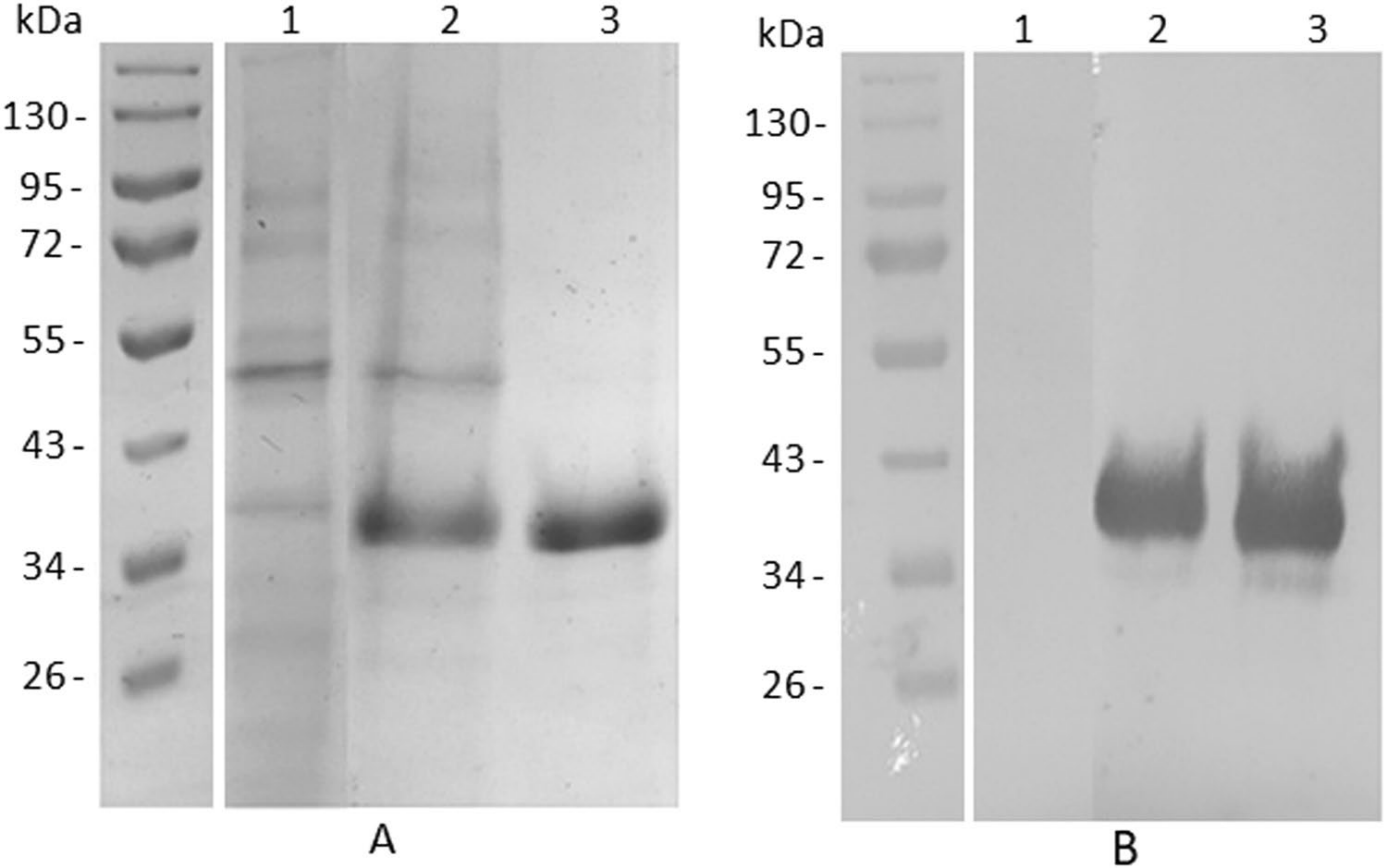
SDS-PAGE (**A**) and Western Blotting (**B**) of AgraGH45-1 expression and purification. (1) Negative control (supernatant of a 5 days culture of *P. pastoris* transformed with empty pGAPZα-B); (2) Supernatant of a 5 days culture of *P. pastoris* transformed with pGAPZα-B_ AgraGH45-1; (3) 4 µg of purified AgraGH45-1. Gel and Blot were cropped to improve the clarity and conciseness of the presentation. Full-length blots/gels are presented in Supplementary Fig. 1.

Around 10 µg of pGAPZα-B/*AgraGH45-1* were used to transform X-33 *P. pastoris* electrocompetent cells according to procedures described in Pichia Expression Kit (Thermo Fisher Scientific, USA). *Pichia pastoris* system is the method of choice for heterologous expression of functional eukaryotic soluble proteins since this yeast is able to perform post-translational modifications, like N-glycosylation, required for protein functional folding^41,42^. β-1,4-Endoglucanase is known to present glycosylation sites (Fig. 3), and such post-translational modification is crucial for its activity^43^. Recently, several β-1,4-Endoglucanase enzymes have been successfully expressed in *P. pastoris*^40,43,44^. The heterologous AgraGH45-1 was purified by affinity chromatography and detected by Western blotting with Anti-6xHis antibody (Fig. 4). The recombinant enzyme was stable for five days under expression conditions (28 °C and 225 rpm). However, even though its predicted molecular weight is around 23 kDa, through our Western blotting detection, it appeared to be ∼35 kDa (Fig. 4). This may be explained by mutation on the Kex2 cleavage site^45^, which could prevent the signal peptide excision after protein secretion. The retention of the α-factor signal peptide could increase the molecular weight of the recombinant protein in 10 kDa. Hiperglycosylation is also commonly known to increase the molecular weight of recombinant proteins expressed in *P. Pastoris*^46^. In addition, according to the parameters for heterologous production of a recombinant enzyme, other properties, like optimal temperature and pH for enzymatic activity, can differ from the native enzyme. Chahed and colleagues produced a GH45 endoglucanase from *Sclerotinia sclerotiorum* that shows maximum activity at pH 7.0 and 60 °C, against pH 5.0 and 60 °C for the native enzyme^46^. In another study, Akbarzadeh and colleagues presented a recombinant endoglucanase II from *Trichoderma reesei* expressed in *P. pastoris* with reduced disulfide bonds. The recombinant enzyme showed higher activity and thermostability than the native enzyme^47^. Such variations could even better adjust the recombinant enzyme for industrial physicochemical conditions for plant biomass conversion.

### Evaluation of pH influence and substrate type on AgraGH45-1 activity

**Figure 5:**
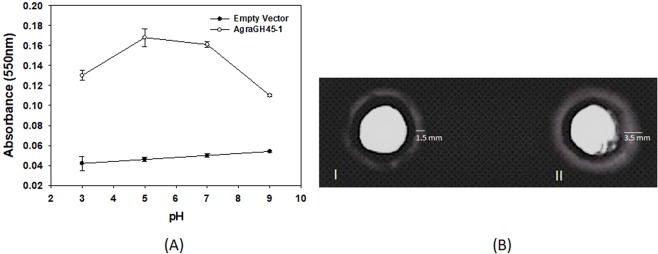
AgraGH45-1 1 enzymatic activity assessment. (**A**) AgraGH45-1 activity at pH ranging from 3.0 to 9.0. The enzymatic reaction was initiated by adding 50 ml of OBR-HEC (5 mg/ml) as substrate. Each point is the average of three measurements. Increase in Abs 550 nm is proportional to substrate degradation. (**B**) AgraGH45-1 activity over carboxymethylcellulose in a radial diffusion assay. (I) Culture of *P. pastoris* transformed with empty vector (50 µL) (negative control). (II) Culture of *P. pastoris* transformed with pGAPZα-B/AgraGH45-1 (50 µL). The white haloes on the dark blue background indicate that activity on B sample is more than 2 folds higher than control.

Despite efforts for making cellulosic biofuels via enzymatic hydrolysis have begun since the 1990s, industry is still looking for new enzyme classes displaying superior hydrolysis kinetics and good stability for commercial purposes^8,9^. The high cost of cellulase enzyme is one of the major barriers for commercialization of bioethanol production from lignocelluloses biomass^10^. The pH and temperature are key parameters to improve enzymatic saccharification step in bioconversion processes^48^. Moreover, the stability of enzymes involved in lignocellulosic degradation, when exposed to such parameters, can contribute to a cost-effective bioethanol production. The major part of commercial cellulases suitable for bioethanol conversion has shown the best activity at pH ranging from 4 to 5 and at temperatures close to 50 °C^49^. More recently, two recombinant β-1,4-Endoglucanases were expressed in *P. pastoris* and evaluated for optimal pH, temperature and stability (AnCel5A – from *A. niger*, and TtCel45A – from thermophilic fungus *Theilavia terrestris*)^40,43^. Both enzymes showed optimal pH and temperature for its activity according to industrial requirements, as also achieved by AgraGH45-1. Nevertheless, AgraGH45-1 seems to keep its higher activity in a wider pH range. The enzymatic activity of AgraGH45-1 over Hydroxyethylcellulose (HEC) substrate was higher at pH 5.0 when analyzed in a range of pH from 3 to 9. Figure 5A shows that AgraGH45-1 efficiency over HEC is substantially stable at pH ranging from 5.0 to 7.0, dropping only 5.5%. On the other hand, the pH range for higher activity of AnCel5A and TtCel45A was from 4.0 to 5.0. AgraGH45-1appears to keep its activity in a wider pH range, notably the range needed for bioethanol bioprocess. Such property could improve its applicability for plant biomass conversion.

AgraGH45-1 capability to digest another substrate analogous to cellulose, Carboxymethylcellulose (CMC), was also evaluated in a radial diffusion assay. The supernatant of the culture inoculated with *P. pastoris* transformed with pGAPZα-B/*AgraGH45-1* was able to digest CMC more than 2-fold higher than the control at pH 5.0 (Fig. 5B). Previous reports also indicated β-1,4-endoglucanase activity in pH ranging from 4.5 to 5.5 for other insects, such as *Diabrotica virgifera virgifera, Apriona germari*^24,50^, and even for the mollusc *Ampullaria crossean*^51^.

### Evaluation of temperature on AgraGH45-1 activity

**Figure 6:**
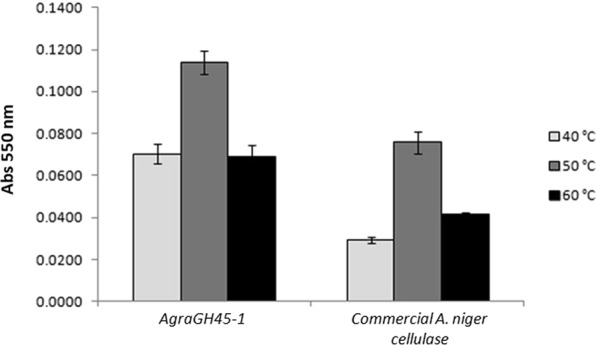
Efficiency of AgraGH45-1 compared to commercial *A. Níger* cellulase. AgraGH45-1 was 1.3-fold more efficient than *A. niger* commercial cellulase to hydrolyze OBR-HEC 5 mg/mL at 50 °C. When analyzed at 40 and 60 °C, the falling ratio of AgraGH45-1 activity was smaller than *A. niger* commercial cellulase.

The efficiency of AgraGH45-1 at pH 5 and temperature ranging from 40 to 60 °C was evaluated and compared to *Aspergillus niger* commercial cellulase over Hydroxyethylcellulose substrate. *A. niger* is the most important source of commercial cellulases and has been used in several industrial process for cellulose bioconversion. Its genome contains around 170 genes involved in polysaccharide degradation^52^. AgraGH45-1 maximum activity was found at 50 °C, and it was 1.3-fold higher than the one from *A. niger* commercial cellulase (Fig. 6). It was also observed that at 40 and 60 °C AgraGH45-1 activity falls 38% and 39%, respectively, compared to its maximum activity, whereas commercial *A. niger* cellulose dropped down 61% and 45%, respectively, compared to its maximum activity at 50 °C. Therefore, these results suggest that AgraGH45-1 is more efficient than *A. niger* commercial cellulase, and its activity can slightly resist to temperature variation. Such AgraGH45-1 property could improve its applicability to an efficient bioprocess for biomass conversion.

The activity of AgraGH45-1 over CMC and HEC demonstrates the capability of that recombinant enzyme to break glycoside bounds in such cellulose analogous substrate. However, it is known that the efficiency of some enzymes over CMC and HEC sometimes is not reproduced over complex biomass substrates^53^. We have now pointed our aims towards further assessing the ability of AgraGH45-1 to degrade complex biomass-derived substrates when added to an enzyme consortium.

In order to improve biofuel production worldwide, advances in agricultural, industrial, and technological areas should contribute to making second generation ethanol production less expensive. Our study attempts to shed a light over such necessity of the renewable fuel industry.

## Conclusion

In this study we performed the cloning of *AgraGH45-1*, an *A. grandis* midgut-specific β-1,4-endoglucanase, as well as assessed both heterologous expression and activity of that recombinant enzyme over substrates analogous to cellulose. Our results strongly indicate that AgraGH45-1 is more efficient than commercial *A. niger* for Hydroxyethylcellulose degradation at 50 °C. In addition, its activity presents smaller variation when assayed at 40 °C and 60 °C than *A. niger* commercial cellulase. AgraGH45-1 can potentially be tested as an enzyme consortium component for plant biomass digestion aiming second-generation ethanol production.

## Methods

### Gene selection

A search for genes coding for plant biomass degradation enzymes from arthropods was performed at the NCBI (National Center for Biotechnology Information - http://www.ncbi.nlm.nih.gov) GenBank. Conserved sequences were used as query in a search for similar sequences within *Anthonomus grandis* transcriptome^30^. An *A. grandis* transcriptome contig harbouring a β-1,4-endoglucanase coding sequence (*A_grandis_454_rep_c954*) was selected by similarity search using the BLASTx tool^54^, based on an e-value threshold of 1e^−30^.

### Insect rearing

Insects were obtained from a colony maintained at the Insect Breeding Laboratory at Embrapa Genetic Resources and Biotechnology in Brasília, DF, Brazil. Eggs, larvae, pupae and adults were kept under controlled temperature conditions (26 ± 2 °C), relative humidity (70 ± 10%) and 12 h:12 h light:dark photo period. Insects were fed daily with artificial diet^55^.

### *A. grandis*

β-1,4-endoglucanase (AgraGH45-1) relative transcripts quantification by RT-qPCR in larva and adult tissues

To verify the expression profile of *AgraGH45-1*, the *A. grandis* carcass and gut of both larvae and adult insects were dissected in 0.15 M NaCl solution. Total RNA was isolated using TRIzol reagent (Thermo Fisher Scientific, USA) according to the manufacturer’s instructions. The integrity of RNA samples was confirmed by 1.0% agarose gel electrophoresis, and a Nanodrop spectrophotometer (Thermo Fisher Scientific, USA) was used to determine RNA quantity. For each sample, 2 μg of total RNA treated with Ambion DNase I (RNase-free) (Thermo Fisher Scientific, USA) was used to synthesize the first-strand cDNA using M-MLV Reverse Transcriptase Kit (Invitrogen, USA) according to the manufacturer’s guidelines. OligoPerfect Designer (Thermo Fisher Scientific, USA) was used to design primers for the reverse transcription quantitative PCR (RT-qPCR). *AgraGH45-1* specific primers and those from the two reference genes (*GAPDH* and *β-tubulin*) used in RT-qPCR are displayed in Table 1. Each RT-qPCR reaction included 5.0 μL of GoTaq qPCR Master Mix 2 × (Promega, USA), 2.6 μL of nuclease-free water, 0.2 μL (0.2 μM) of forward and reverse primers and 2.0 μL of cDNA (diluted 1:20), for a total volume of 10 μL. RT-qPCR assays were performed in a CFX96 thermocycler (Bio-Rad, USA) under the following conditions: incubation at 95 °C for 20 s, followed by 40 cycles at 95 °C for 3 s and 60 °C for 30 s. Reactions were set-up in 96-well Microseal PCR plates (Bio-Rad) in triplicate. At the end of each RT-qPCR experiment a dissociation curve for each amplicon was created to verify the possible formation of primer dimers or sample contamination. The efficiency of each primer for each reaction and the Cq value were calculated individually using the qPCR miner software (www.miner.ewindup.info)^56^. Analyses of *AgraGH45-1* expression were performed by using qBASE + software (Biogazelle, Belgium)^57^.

### *AgraGH45-1* cloning and insertion into expression vector

Nucleotide sequence from the selected contig was used to design primers to clone, firstly the whole *AgraGH45-1* ORF sequence. The *AgraGH45-1* was amplified from total RNA extracts of *A. grandis* by 3′ RACE-PCR and cloned in pGEM-T Easy Vector (Promega, USA). Then, a new primer set was designed to subclone *AgraGH45-1* lacking the signal peptide correspondent sequence. The signal peptide sequence was identified using Signal-P tool^58^. Forward primer was designed to insert an *Eco*RI site (underlined) at the 5′ end of *AgraGH45-1* (*AgraGH451GAPfwd*: 5′ TAAGAATTCCACTTAGCGGTTCTGGAACTACT 3′). Reverse primer was designed to insert a *Sal*I site (underlined) at the 3′ end of *AgraGH45-1* (*AgraGH451GAPrev*: 5′ TTAGTCGACCATATTGTCATTTACACAACC 3′). The amplified material, with new primer set, from pGEM-T-AgraGH45-1 clone was subcloned again in pGEM-T Easy, and named pGEM-T-AgraGH45-1ΔSP. This clone was digested with *Eco*RI and *Sal*I, the insert was purified, and then inserted into pGAPZα-B expression vector previously digested with the same restriction enzymes, resulting in pGAPZα-B-AgraGH45-1ΔSP clone. After all cloning procedures a *de novo* nucleotide sequencing was performed to confirm the correct insertion in frame with vector start codon.

### Alignment of the amino acid sequence of AgraGH45-1 with others beetle cellulases

Nucleotide sequence of *AgraGH45-1* was translated into its amino acid sequence using Expasy Translate tool (https://web.expasy.org/translate/) and aligned with Oa-EGase II (a β-1,4-endoglucanase from *O. albomarginata* [GenBank:GU001942]), and β-1,4-endoglucanase from *R. ferrugineus* (Rf- GH45) [GenBank: ANU06045.1]. Sequences alignment was performed with Clustal Omega tool for the multiple alignment of sequences^59^ using default parameters and manually adjusted accordingly.

### Heterologous expression of AgraGH45-1

The expression vector harbouring the *AgraGH45-1* (pGAPZα-B-AgraGH45-1 ΔSP) was linearized with *Avr*II restriction enzyme and used to transform X-33 *P. pastoris* electrocompetent cells according to the Pichia Expression Kit (Thermo Fisher Scientific, USA). Transformed cells were streaked in YPG (1% Yeats Extract, 2% Peptone and 2% Glucose) medium containing Zeocine 100 µg/mL and kept at 28 °C for four days. Emerging colonies were screened by PCR using specific primers. PCR positives colonies were selected for small scale expression assay in liquid YPG/Zeocine medium (50 mL of medium, 28 °C at 225 rpm for three days). A colony transformed with pGAPZα-B empty vector was used as negative control during expression assays.

### Evaluation of pH influence on AgraGH45-1 activity

AgraGH45-1 activity over Hydroxyethylcellulose conjugated to Ostazin Brilliant-Red (OBR-HEC) chromophore^60^ was evaluated on a range of pH. Therefore, 25 µL of the AgraGH45-1 expression supernatant was incubated for 15 min at 45 °C with different buffers (50 mM Sodium Citrate pH 3.0, 50 mM Sodium Acetate pH 5.0, 50 mM Sodium Phosphate pH 7.0 and 50 mM Tris/HCl pH 9.0). The reaction was started by addition of 50 µL of OBR-HEC 5 mg/mL in a final volume of 150 µL and kept for 30 min at 45 °C. Reaction was stopped by adding 900 µl of ethanol and then centrifuged at 10,000 × g. The supernatant absorbance was read at 550 nm. As a control for each reaction was used 25 µL of the supernatant from an expression inoculated with *P. pastoris* transformed with an empty pGAPZα-B vector. Each reaction was carried out in triplicate.

### Radial diffusion assay

Activity of the recombinant enzyme was detected as described by Jimenez and colleagues^24^. Plates were prepared with agarose (1%), Congo red (0,02%) and Carboxymetylcelullose (CMC) (0,5%) dissolved in 50 mM sodium acetate pH 5.0. After solidification into Petri plates, circular wells were punched into the agar with a 5 mm diameter cork borer to a depth of 10 mm. Activity assay was performed with 50 µL of cell culture supernatant inoculated with *P. pastoris* transformed with pGAPZα-B-AgraGH45-1 ΔSP and incubated at 45 °C overnight. After 24 hours the residual Congo red dye was removed by rinsing the plate with distilled water and then fixed by flooding the plate with acetic acid (10%) for 1 hour at room temperature. β-1,4-endoglucanase activity zones appeared as white haloes on a dark blue background. As a negative control it was used 50 µL of cell culture supernatant inoculated with *P. pastoris* transformed with empty pGAPZα-B.

### Purification of AgraGH45-1

A X-33 *P. pastoris* colony transformed with pGAPZα-B-AgraGH45-1 ΔSP was inoculated in 100 mL of YPG/Zeocine 100 µg/mL and kept at 28 °C and 225 rpm for 3 days. After that, 10 mL of the culture was used to inoculate 1 L of YPG/Zeocine 100 µg/mL. The culture was kept at 28 °C and 225 rpm for 4 days. The cells were harvested by centrifugation, the supernatant filtered through 0.2 µm and then diluted in sodium phosphate buffer pH 7.4 (1:1) to a final concentration of 20 mM. Sodium chloride was added to a final concentration of 0.5 M. The final volume was kept circulating through a HisTrap FF crude 5 mL (G.E. Healthcare) column previously equilibrated with binding buffer (20 mM sodium phosphate pH 7.4, 0.5 M NaCl). The column was washed with 6 volumes of binding buffer containing 20 mM imidazole, and the recombinant AgraGH45-1 was eluted with binding buffer containing 0.5 M imidazole. Eluted fractions were dialyzed against 0.25 mM sodium bicarbonate and freeze-dried. Protein samples were quantified and used in electrophoretic assays to molecular weight determination. AgraH45-1 was detected by Western Blotting with 6 × -His Tag Monoclonal Antibody (Thermo Fisher Scientific, USA).

### Comparison of AgraGH45-1 activity and thermo stability with *Aspergillus niger* commercial cellulase

AgraGH45-1 and commercial *A. niger* cellulase (Sigma Aldrich, USA) activity on Hydroxyethylcellulose conjugated to Ostazin Brilliant-Red (OBR-HEC) was evaluated at pH 5.0 and temperature ranging from 40 to 60 °C. Therefore, 10 µg of both enzymes were dissolved in 25 µL of 50 mM sodium citrate pH 5.0 and incubated for 15 min at 45 °C. The reaction was started by addition of 50 µL of OBR-HEC 5 mg/mL in a final volume of 150 µL and kept for 30 min at 40 °C. The same procedure was repeated to incubation at 50 and 60 °C. Reactions were stopped by adding 900 µl of ethanol and then centrifuged at 10,000 × g. The supernatant absorbance was read at 550 nm. Each reaction was carried out in triplicate.

## Supplementary information

Supplementary Information
