# Author Correction: Enzymatic activity of a recombinant β-1,4-endoglucanase from the Cotton Boll Weevil (*Anthonomus grandis*) aiming second generation ethanol production

**DOI:** 10.1038/s41598-020-62217-2

**Published:** 2020-03-19

**Authors:** Liz Nathalia Ibarra, Ana Elizabeth Oliveira de Araújo Alves, José Dijair Antonino, Guilherme Souza Prado, Clidia Eduarda Moreira Pinto, Carlos Ricardo Soccol, Érico Augusto Rosas de Vasconcelos, Maria Fátima Grossi-de-Sa

**Affiliations:** 1 Universidade Federal do Paraná – UFPR, Curitiba, PR 81530-980 Brazil; 2 Embrapa Recursos Genéticos e Biotecnologia, Brasília, DF 70770-917 Brazil; 3 Universidade de Brasília – UnB, Biology Institute, Brasília, DF 70910-900 Brazil; 4 Universidade Federal Rural de Pernambuco – UFRPE, Recife-PE, 52171-900 Brazil; 5 Universidade Católica de Brasília – UCB, Brasília, DF 70790-160 Brazil; 6 Centro Universitário Euroamericano - UNIEURO, Brasilia, DF 70200-001 Brazil

Correction to: *Scientific Reports* 10.1038/s41598-019-56070-1, published online 20 December 2019

The original version of this Article contained a typographical error in the spelling of the author Ana Elizabeth Oliveira de Araújo Alves, which was incorrectly given as Ana Nathalia Oliveira de Araújo Alves.

This error has now been corrected in the PDF and HTML versions of the Article.

