# Supplementary figures and images for: Enzymatic activity of a recombinant β-1,4-endoglucanase from the Cotton Boll Weevil (Anthonomus grandis) aiming second generation ethanol production
Source: Sci Rep. 2019 Dec 20;9:19580. doi: 10.1038/s41598-019-56070-1 (PMC6925290; doi:10.1038/s41598-019-56070-1)

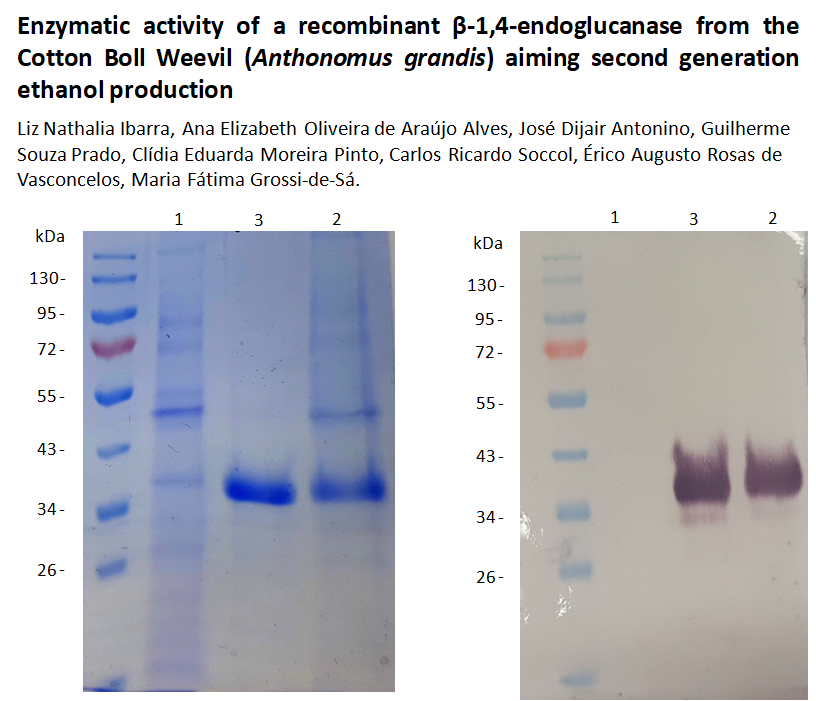

Supplement: Supplementary file 1 — Supplementary Information [file 41598_2019_56070_MOESM1_ESM.png]
